# Retraction Note: An efficient hybrid stock trend prediction system during COVID-19 pandemic based on stacked-LSTM and news sentiment analysis

**DOI:** 10.1007/s11042-026-21614-x

**Published:** 2026-04-25

**Authors:** Marwa Sharaf, Ezz El-Din Hemdan, Ayman El-Sayed, Nirmeen A. El-Bahnasawy

**Affiliations:** Computer Science and Engineering Department, Faculty of Electronic Engineering, Menoufia University, Menouf, Egypt

**Retraction Note: Multimedia Tools and Applications (2022) 82:23945–23977**

10.1007/s11042-022-14216-w

The Publisher has retracted this article in agreement with the Editor-in-Chief. Following publication, this article was found to contain multiple instances of non-standard phrases, also known as tortured phrases, in place of expected well-established phrases [1]. The Editor-in-Chief therefore no longer has confidence in the reliability and provenance of this article.

The authors have not responded to correspondence regarding this retraction. 
